# 4,7,8-Trimethyl-2*H*-chromen-2-one

**DOI:** 10.1107/S1600536812009646

**Published:** 2012-03-10

**Authors:** Jian-Xin Yang, Xue-Mei Tan, Xiang-Hui Wang, Yin Wang

**Affiliations:** a Institute of Materials and Chemical Engineering, Hainan University, Haikou 570228, People’s Republic of China; b Institute of Environmental Science and Engineering, Kunming University of Science and Technology, Kunming 650093, People’s Republic of China

## Abstract

The mol­ecule of the title compound, C_12_H_12_O_2_, is essentially planar, with a maximum deviation from the mean plane of all non-H atoms of 0.038 (1) Å for the methyl C atom in the 8-position. The crystal structure is characterized by anti­parallel π–π stacking along the *c* axis, with centroid–centroid distances as short as 3.866 (1) Å. In the crystal, C—H⋯O hydrogen bonds connect the mol­ecules across the stacks into ribbons in the *a*-axis direction.

## Related literature

For general background to the pharmacological activity of coumarin derivatives, see: Xie *et al.* (2001 ▶); Tanitame *et al.* (2004 ▶); Shao *et al.* (1997 ▶); Rendenbach-Müller *et al.* (1994 ▶); Pochet *et al.* (1996 ▶). For a related structure, see: Gowda *et al.* (2010 ▶).
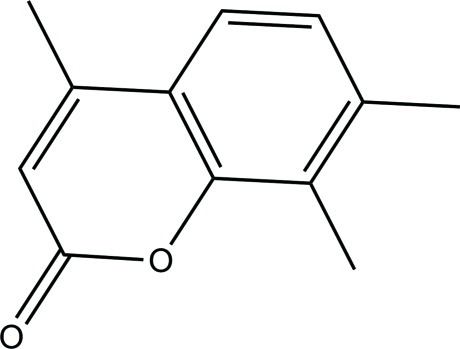


## Experimental

### 

#### Crystal data

C_12_H_12_O_2_
*M* *_r_* = 188.22
Monoclinic, 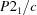

*a* = 7.276 (3) Å
*b* = 18.075 (6) Å
*c* = 7.246 (3) Å
β = 97.055 (5)°
*V* = 945.8 (6) Å^3^
*Z* = 4
Mo *K*α radiation
μ = 0.09 mm^−1^
*T* = 153 K
0.44 × 0.31 × 0.26 mm

#### Data collection

Rigaku AFC10/Saturn724+ diffractometer
8545 measured reflections
2747 independent reflections
2176 reflections with *I* > 2σ(*I*)
*R*_int_ = 0.028

#### Refinement

*R*[*F* ^2^ > 2σ(*F* ^2^)] = 0.050
*wR*(*F* ^2^) = 0.112
*S* = 1.00
2747 reflections
130 parameters
H-atom parameters constrained
Δρ_max_ = 0.28 e Å^−3^
Δρ_min_ = −0.33 e Å^−3^

### 

Data collection: *CrystalClear* (Rigaku, 2008 ▶); cell refinement: *CrystalClear*; data reduction: *CrystalClear*; program(s) used to solve structure: *SHELXS97* (Sheldrick, 2008 ▶); program(s) used to refine structure: *SHELXL97* (Sheldrick, 2008 ▶); molecular graphics: *SHELXTL* (Sheldrick, 2008 ▶) and *OLEX2* (Dolomanov *et al.*, 2009 ▶); software used to prepare material for publication: *SHELXTL* and *publCIF* (Westrip, 2010 ▶).

## Supplementary Material

Crystal structure: contains datablock(s) I, global. DOI: 10.1107/S1600536812009646/ld2048sup1.cif

Structure factors: contains datablock(s) I. DOI: 10.1107/S1600536812009646/ld2048Isup2.hkl

Supplementary material file. DOI: 10.1107/S1600536812009646/ld2048Isup3.cml

Additional supplementary materials:  crystallographic information; 3D view; checkCIF report

## Figures and Tables

**Table 1:** Hydrogen-bond geometry (Å, °)

*D*—H⋯*A*	*D*—H	H⋯*A*	*D*⋯*A*	*D*—H⋯*A*
C2—H2⋯O2^i^	0.95	2.56	3.460 (2)	159
C10—H10*C*⋯O2^ii^	0.98	2.54	3.493 (2)	164

Symmetry codes: (i) 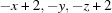; (ii) 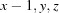.

**Fig. 1.**
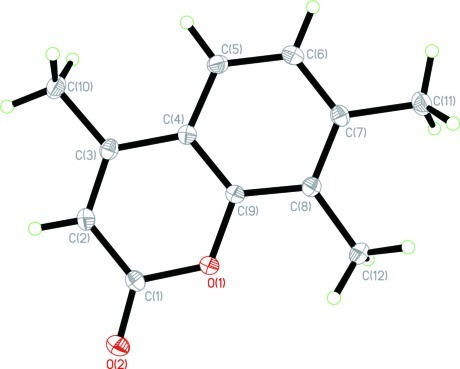
The molecular structure of the title compound with the atom numbering scheme. Displacement ellipsoids are drawn at the 30% probability level. H atoms are presented as a small spheres of arbitrary radius.

**Fig. 2.**
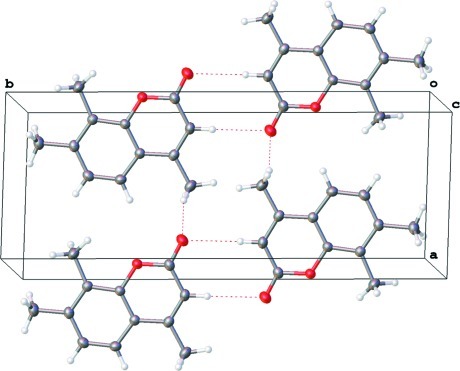
H-bonding in the crystals of the title compound. Intermolecular hydrogen bonds are shown as dashed lines.

**Fig. 3.**
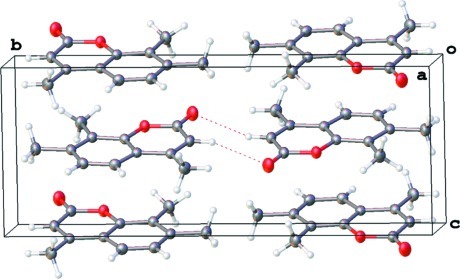
π–π stacking in the crystal of te title compound.

## supplementary crystallographic information

### Comment

Coumarin derivatives exhibit a wide variety of pharmacological activities including anti-HIV (Xie *et al.*, 2001), antibacterial (Tanitame *et al.*, 2004), antioxidant (Shao *et al.*, 1997), antithrombotic (Rendenbach-Müller *et al.*, 1994) and antiinflammatory (Pochet *et al.*, 1996) activities.

The molecular structure is shown in Fig. 1. In the crystal the molecules are linked by C—H···O hydrogen bonds to form ribbon-like motives (Table 1 and Fig. 2).

### Experimental

2,3-Dimethyl phenol (10.50 mmol) was slowly added at 278–288 K to a mixture of *para*-toluenesulfonic acid (0.5 g) and acetylacetic ester (10.50 mmol) while stirring for 30 min. The reaction mixture was stirred continuously for 12 more hours at room temperature and then poured into ice–water mixture (100 ml). The obtained solid was filtered off, washed with cold water and dried at room temperature. Colorless crystals of the title compound suitable for X-ray structure analysis were obtained by slow evaporation of a solution in the mixture of ethanol/ether over a period of two days.

### Refinement

H atoms were placed in calculated positions with C—H = 0.93 (aromatic) and 0.96 Å (methyl), and refined in riding mode with *U*_iso_(H) = 1.2U_eq_(C) (aromatic) and *U*_iso_(H) = 1.5U_eq_(C) (methyl). The positions of the methyl H atoms were optimized rotationally.

### Crystal data

**Table:**

C_12_H_12_O_2_	*F*(000) = 400
*M**_r_* = 188.22	*D*_x_ = 1.322 Mg m^−^^3^
Monoclinic, *P*2_1_/*c*	Mo *K*α radiation, λ = 0.71073 Å
*a* = 7.276 (3) Å	Cell parameters from 3283 reflections
*b* = 18.075 (6) Å	θ = 2.3–30.0°
*c* = 7.246 (3) Å	µ = 0.09 mm^−^^1^
β = 97.055 (5)°	*T* = 153 K
*V* = 945.8 (6) Å^3^	Prism, colorless
*Z* = 4	0.44 × 0.31 × 0.26 mm

### Data collection

**Table:**

Rigaku AFC10/Saturn724+ diffractometer	2176 reflections with *I* > 2σ(*I*)
Radiation source: Rotating Anode	*R*_int_ = 0.028
Graphite monochromator	θ_max_ = 30.1°, θ_min_ = 3.1°
Detector resolution: 28.5714 pixels mm^-1^	*h* = −9→10
phi and ω scans	*k* = −24→25
8545 measured reflections	*l* = −10→10
2747 independent reflections	

### Refinement

**Table:**

Refinement on *F*^2^	Primary atom site location: structure-invariant direct methods
Least-squares matrix: full	Secondary atom site location: difference Fourier map
*R*[*F*^2^ > 2σ(*F*^2^)] = 0.050	Hydrogen site location: inferred from neighbouring sites
*wR*(*F*^2^) = 0.112	H-atom parameters constrained
*S* = 1.00	*w* = 1/[σ^2^(*F*_o_^2^) + (0.0269*P*)^2^ + 0.551*P*] where *P* = (*F*_o_^2^ + 2*F*_c_^2^)/3
2747 reflections	(Δ/σ)_max_ = 0.001
130 parameters	Δρ_max_ = 0.28 e Å^−^^3^
0 restraints	Δρ_min_ = −0.33 e Å^−^^3^

### Special details

**Table:**

Geometry. All e.s.d.'s (except the e.s.d. in the dihedral angle between two l.s. planes) are estimated using the full covariance matrix. The cell e.s.d.'s are taken into account individually in the estimation of e.s.d.'s in distances, angles and torsion angles; correlations between e.s.d.'s in cell parameters are only used when they are defined by crystal symmetry. An approximate (isotropic) treatment of cell e.s.d.'s is used for estimating e.s.d.'s involving l.s. planes.
Refinement. Refinement of *F*^2^ against ALL reflections. The weighted *R*-factor *wR* and goodness of fit *S* are based on *F*^2^, conventional *R*-factors *R* are based on *F*, with *F* set to zero for negative *F*^2^. The threshold expression of *F*^2^ > σ(*F*^2^) is used only for calculating *R*-factors(gt) *etc*. and is not relevant to the choice of reflections for refinement. *R*-factors based on *F*^2^ are statistically about twice as large as those based on *F*, and *R*- factors based on ALL data will be even larger.

### Fractional atomic coordinates and isotropic or equivalent isotropic displacement parameters (Å^2^)

**Table:**

	*x*	*y*	*z*	*U*_iso_*/*U*_eq_	
H2	0.8364	0.0375	0.9962	0.029*	
H5	0.4346	0.2356	0.8389	0.030*	
H6	0.4708	0.3629	0.8497	0.030*	
H10A	0.5336	0.0381	0.8616	0.035*	
H10B	0.4726	0.1097	0.7392	0.035*	
H10C	0.4183	0.1026	0.9459	0.035*	
H11A	0.6432	0.4698	0.9153	0.037*	
H11B	0.8581	0.4625	0.8914	0.037*	
H11C	0.7941	0.4634	1.0949	0.037*	
H12A	1.1505	0.3139	1.1756	0.036*	
H12B	1.0632	0.3953	1.1693	0.036*	
H12C	1.1483	0.3633	0.9921	0.036*	
C1	1.01364 (18)	0.12436 (7)	1.07922 (18)	0.0243 (3)	
C2	0.84235 (18)	0.08992 (7)	1.00252 (18)	0.0244 (3)	
C3	0.69054 (18)	0.12937 (7)	0.93951 (18)	0.0224 (3)	
C4	0.69972 (17)	0.20939 (7)	0.94548 (17)	0.0207 (2)	
C5	0.55137 (18)	0.25623 (7)	0.88512 (19)	0.0248 (3)	
C6	0.57298 (19)	0.33200 (7)	0.89207 (19)	0.0251 (3)	
C7	0.74262 (18)	0.36425 (7)	0.96038 (18)	0.0234 (3)	
C8	0.89411 (18)	0.31925 (7)	1.02295 (18)	0.0221 (3)	
C9	0.86750 (17)	0.24271 (7)	1.01382 (17)	0.0206 (2)	
C10	0.51347 (19)	0.09170 (8)	0.8651 (2)	0.0296 (3)	
C11	0.7611 (2)	0.44726 (7)	0.9660 (2)	0.0305 (3)	
C12	1.08022 (19)	0.35066 (8)	1.0964 (2)	0.0299 (3)	
O1	1.02050 (12)	0.20031 (5)	1.07807 (13)	0.0240 (2)	
O2	1.15405 (14)	0.09227 (6)	1.14331 (15)	0.0338 (3)	

### Atomic displacement parameters (Å^2^)

**Table:**

	*U*^11^	*U*^22^	*U*^33^	*U*^12^	*U*^13^	*U*^23^
C1	0.0259 (6)	0.0213 (6)	0.0259 (6)	0.0034 (5)	0.0042 (5)	−0.0003 (5)
C2	0.0277 (7)	0.0189 (6)	0.0268 (6)	−0.0011 (5)	0.0041 (5)	−0.0012 (5)
C3	0.0246 (6)	0.0225 (6)	0.0205 (6)	−0.0035 (5)	0.0040 (5)	−0.0007 (5)
C4	0.0213 (6)	0.0211 (6)	0.0200 (6)	−0.0004 (4)	0.0038 (5)	−0.0002 (5)
C5	0.0202 (6)	0.0273 (6)	0.0262 (6)	−0.0003 (5)	0.0001 (5)	0.0003 (5)
C6	0.0238 (6)	0.0258 (6)	0.0254 (6)	0.0048 (5)	0.0014 (5)	0.0024 (5)
C7	0.0276 (6)	0.0204 (6)	0.0223 (6)	0.0014 (5)	0.0039 (5)	0.0018 (5)
C8	0.0223 (6)	0.0222 (6)	0.0220 (6)	−0.0010 (5)	0.0028 (5)	0.0000 (5)
C9	0.0196 (6)	0.0209 (6)	0.0214 (6)	0.0018 (4)	0.0025 (5)	0.0007 (5)
C10	0.0283 (7)	0.0267 (7)	0.0329 (7)	−0.0080 (5)	0.0006 (6)	−0.0005 (6)
C11	0.0366 (8)	0.0209 (6)	0.0335 (8)	0.0024 (5)	0.0016 (6)	0.0023 (5)
C12	0.0265 (7)	0.0257 (7)	0.0359 (7)	−0.0052 (5)	−0.0021 (6)	−0.0010 (6)
O1	0.0205 (4)	0.0212 (4)	0.0296 (5)	0.0020 (3)	−0.0004 (4)	0.0000 (4)
O2	0.0287 (5)	0.0271 (5)	0.0439 (6)	0.0075 (4)	−0.0030 (4)	−0.0006 (4)

### Geometric parameters (Å, º)

**Table:**

C1—C2	1.4421 (19)	C8—C12	1.5042 (18)
C2—H2	0.9500	C10—H10A	0.9800
C2—C3	1.3465 (18)	C10—H10B	0.9800
C3—C4	1.4484 (18)	C10—H10C	0.9800
C3—C10	1.4979 (18)	C11—H11A	0.9800
C4—C5	1.3990 (18)	C11—H11B	0.9800
C4—C9	1.3964 (17)	C11—H11C	0.9800
C5—H5	0.9500	C12—H12A	0.9800
C5—C6	1.3788 (19)	C12—H12B	0.9800
C6—H6	0.9500	C12—H12C	0.9800
C6—C7	1.3994 (19)	O1—C1	1.3739 (16)
C7—C8	1.3998 (18)	O1—C9	1.3842 (15)
C7—C11	1.5067 (19)	O2—C1	1.2163 (16)
C8—C9	1.3973 (18)		
			
O1—C1—C2	117.34 (11)	C9—C8—C12	120.26 (12)
O2—C1—C2	125.95 (13)	C4—C9—C8	123.63 (11)
O2—C1—O1	116.71 (12)	O1—C9—C4	120.83 (11)
C1—C2—H2	118.8	O1—C9—C8	115.54 (11)
C3—C2—H2	118.8	H10A—C10—H10B	109.5
C3—C2—C1	122.43 (12)	H10A—C10—H10C	109.5
C2—C3—C4	119.07 (12)	H10B—C10—H10C	109.5
C2—C3—C10	120.99 (12)	C3—C10—H10A	109.5
C4—C3—C10	119.94 (12)	C3—C10—H10B	109.5
C5—C4—C3	124.33 (12)	C3—C10—H10C	109.5
C9—C4—C3	118.45 (11)	H11A—C11—H11B	109.5
C9—C4—C5	117.21 (12)	H11A—C11—H11C	109.5
C4—C5—H5	119.7	H11B—C11—H11C	109.5
C6—C5—H5	119.7	C7—C11—H11A	109.5
C6—C5—C4	120.63 (12)	C7—C11—H11B	109.5
C5—C6—H6	119.4	C7—C11—H11C	109.5
C5—C6—C7	121.22 (12)	H12A—C12—H12B	109.5
C7—C6—H6	119.4	H12A—C12—H12C	109.5
C6—C7—C8	119.86 (12)	H12B—C12—H12C	109.5
C6—C7—C11	119.76 (12)	C8—C12—H12A	109.5
C8—C7—C11	120.39 (12)	C8—C12—H12B	109.5
C7—C8—C12	122.29 (12)	C8—C12—H12C	109.5
C9—C8—C7	117.45 (12)	C1—O1—C9	121.80 (10)

### Hydrogen-bond geometry (Å, º)

**Table:**

*D*—H···*A*	*D*—H	H···*A*	*D*···*A*	*D*—H···*A*
C2—H2···O2^i^	0.95	2.56	3.460 (2)	159
C10—H10*C*···O2^ii^	0.98	2.54	3.493 (2)	164

Symmetry codes: (i) −*x*+2, −*y*, −*z*+2; (ii) *x*−1, *y*, *z*.
